# Tumor associated PD-L1 expression pattern in microscopically tumor positive sentinel lymph nodes in patients with melanoma

**DOI:** 10.1186/s12967-015-0678-7

**Published:** 2015-09-30

**Authors:** Ahmad A. Tarhini, Haris Zahoor, Jennifer H. Yearley, Christopher Gibson, Zahra Rahman, Rachel Dubner, Uma N. M. Rao, Cindy Sander, John M. Kirkwood

**Affiliations:** University of Pittsburgh, Pittsburgh, PA USA; University of Pittsburgh Cancer Institute, 5150 Centre Avenue, Pittsburgh, PA 15232 USA; Merck, Philadelphia, PA USA

**Keywords:** Melanoma, PD-1, PD-L1, Sentinel lymph node

## Abstract

**Background:**

Characterization of PD-L1 expression within clinically/radiologically negative but microscopically tumor positive sentinel lymph nodes (SLN) is important to our understanding of the relevance of this immune checkpoint pathway for adjuvant therapy.

**Methods:**

Patients included had primary cutaneous melanoma, Breslow thickness of 2.01–4.0 or >4 mm with or without tumor ulceration (T3a, T3b, T4a, T4b). All patients had microscopically tumor positive SLN. Hematoxylin and eosin (H&E) staining was performed, followed by PD-L1 immunohistochemical (IHC) staining using a preliminary IHC assay with anti-PD-L1 antibody clone 22C3. The slides were separately evaluated by two pathologists (JY and CG). Samples containing metastatic melanoma lesions were scored separately for PD-L1 expression in intratumoral and peritumoral locations, by utilizing two scoring methods.

**Results:**

Twenty-four patients where metastatic melanoma presence in the SLN was confirmed by H&E review of the cut sections were included in the final analysis of PD-L1 expression. SLN tumor size ranged from 1 to 2 mm. For three patients, the melanin content was too high to confidently assign a PD-L1 score. For the remaining 21 patients, all had some evidence of either intratumoral or peritumoral PD-L1 expression. The frequency of intratumoral tumor-associated PD-L1 expression was: 0 % of tumor cells (3 pts, 14 %); <1 % (5 pts, 24 %); 1–10 % (6 pts, 29 %) and >10 % (7 pts, 33 %).

**Conclusions:**

Tumor-associated PD-L1 expression is readily detectable within melanoma micrometastases in the SLN of the majority of patients. These results support the testing of a therapeutic role for PD1/PD-L1 inhibition in the adjuvant setting, targeting melanoma micrometastases.

## Background

PD-1 is an immune-inhibitory receptor belonging to CD28/CTLA4 receptor family that is expressed on activated T cells, B cells and monocytes [1, 2]. PD-1 is also expressed on T regulatory cells where it interacts with dendritic cells and NK T cells, and shown to be associated with anergy and tumor immune escape [3–5]. The role of PD-1 as a negative regulator of T cell activity is mediated through its interaction with its ligands PD-L1 and PD-L2 (also known as B7-H1 and B7-H2 based on the similarity to other B7 family molecules) that are expressed on immune cells and tumor cells [6]. PD-L1 is expressed on T and B cells, macrophages and dendritic cells [7]. PD-L1 and PD-L2 are expressed on many human tumors including melanoma, glioblastoma, non-small cell lung cancer and urothelial, ovarian, breast, cervical, colon, pancreatic and gastric carcinoma [8–18]. PD-L1 has been implicated in tumor immune escape from the host immune system and in mediating tumor anti-apoptotic activity [2, 19–21]. PD-1 ligand 1 and 2 (PD-Ls) expressed on antigen-presenting cells have been shown to indirectly induce T cell anergy or exhaustion via PD-1 on T cells, whereas PD-L1 expressed on peripheral tissues directly suppresses self-reactive lymphocytes [22, 23]. PD-Ls expressed on tumors regulate the generation of adaptive regulatory T cells resulting in tumor-induced immune suppression [5], including the suppression of the effector function of CD8+ T cells [21]. Interestingly, a significant inverse correlation was observed between PD-L1 expression and the intraepithelial CD8+ T lymphocyte count, suggesting that PD-L1 on tumor cells directly suppresses antitumor CD8+ T cells [9]. PD-1 blockade has been shown to enhance the expansion and functional capacity of human melanoma antigen specific cytotoxic T cells [24]. Clinically, higher expression levels of PD-L1 on tumors have been shown to correlate with poor prognosis in several malignant tumors including melanoma, esophagus, kidney, lung, and brain, pancreatic, ovarian and head and neck [8, 9, 12, 16, 25, 26]. These data illustrate a central role for the PD-1/PD-L1 axis in tumor immune escape and have led to the clinical targeting of PD-1 and PD-L1 as an antitumor strategy.

Pembrolizumab and nivolumab, both of which target PD-1, have been approved for the treatment of metastatic melanoma. Approval of pembrolizumab was granted based on data from a cohort of a phase I trial (KEYNOTE-002) in which 411 patients with advanced melanoma who were refractory to ipilimumab or were ipilimumab naive received pembrolizumab at 2 or 10 mg/kg every 3 weeks or 10 mg/kg every 2 weeks [27, 28]. Overall response rates were 40 and 28 % in ipilimumab-naive and ipilimumab-refractory treatment arms, respectively. In addition, response rates and progression-free survival were significantly higher in patients with high PD-L1 tumor expression compared with patients who were considered PD-L1 negative [29]. In December 2014, nivolumab became the second monoclonal antibody targeting the PD-1 receptor to be approved by FDA for the treatment of patients with unresectable or metastatic melanoma and disease progression following ipilimumab and a BRAF inhibitor [30]. Approval for nivolumab was based on results from the first 120 patients enrolled in a phase III trial (CheckMate 037) testing nivolumab versus either dacarbazine or carboplatin/paclitaxel in patients with metastatic melanoma who progressed on or after anti-CTLA-4 therapy and a BRAF inhibitor (if *BRAF* V600 mutation positive). The overall tumor response rates were 32 and 11 % in favor of nivolumab [31]. The use of nivolumab in previously untreated metastatic patients has also shown excellent activity; objective response rate of 40.0 % as compared to 13.9 % in the dacarbazine group [32].

The significant clinical activity of anti-PD1 antibodies has supported their planned testing as adjuvant therapy in patients with operable melanoma at high risk for relapse and death from melanoma. Adjuvant therapy targets micrometastatic disease which is the source of future mortality from melanoma recurrence and presents an opportunity for curing this disease. We hypothesized that micrometastatic tumors that are the source of future melanoma relapse in high risk patients express PD-L1 making them susceptible to PD1/PD-L1 therapeutic blockade. Characterization of PD-L1 expression within clinically/radiologically negative but microscopically tumor positive sentinel lymph nodes (SLN) is important to our understanding of the relevance of this immune checkpoint pathway for adjuvant therapy. In this report, we present data which shows that tumor-associated PD-L1 expression is readily detectable within melanoma micrometastases in the SLN.

## Methods

### Patients

Twenty-four patients with primary cutaneous melanoma were included in this study. All patients had a primary tumor Breslow thickness of 2.01–4.00 mm without (T3a) or with ulceration (T3b), or >4 mm without (T4a) or with ulceration (T4b). Patients had known microscopically tumor positive SLN detected during standard SLN biopsy procedures. All patients provided a written informed consent. Table 1 summarizes patient demographics and baseline disease characteristics.

**Table 1:** Patient demographics and baseline disease characteristics (N = 24 patients)

Variable	No. of patients (%)
Age, years; median (range)	58 (18–75)
Cutaneous primary	24 (100)
Gender	
Female	12 (50)
Male	12 (50)
Performance status (ECOG)	
0	18 (75)
1	6 (25)
AJCC stage	
IIIA	5 (21)
IIIB	16 (67)
IIIC	3 (12)
Ulceration of primary	
Yes	18 (75)
No	6 (25)

*ECOG* Eastern Cooperative Oncology Group, *AJCC* American Joint Committee on Cancer

### Procedures

Cut sections (5 µm) were obtained from formalin-fixed, paraffin-embedded (FFPE) SLN tissue from patients enrolled on this study. Slides were first stained with haematoxylin and eosin. PD-L1 immunostaining was performed using a preliminary immunohistochemistry (IHC) assay with anti-PD-L1 antibody clone 22C3. Slides from two patients were also stained using an anti-HMB45/MelA protocol to better ascertain the presence and/or localization of melanoma lesions in the tissue in order to facilitate interpretation of the PD-L1 staining in those samples. All staining was performed on Dako autostainers at Merck Research Laboratories, Palo Alto, CA. The anti-PD-L1 antibody clone 22C3 is a mouse anti-human PD-L1 IgG1k generated through murine immunization with a fusion protein containing the human extra cellular domain of PD-L1 and subsequent hybridoma formation [33].

The slides were separately evaluated by two pathologists. Samples containing metastatic melanoma lesions were scored separately for PD-L1 expression in intratumoral (including along tumor periphery but with clear tumor cell labeling) and peritumoral (expression external to tumor nodule in immediately surrounding tissue) locations. PD-L1 positivity was defined as partial or complete membrane staining of a tumor cell using the 22C3 antibody [33]. Two scoring methods were utilized: (1) semi-quantitative scoring method—samples containing metastatic melanoma lesions were scored separately for PD-L1 expression in intratumoral (including along tumor periphery but with clear tumor cell labeling) and peritumoral (expression external to tumor nodule in immediately surrounding tissue; immune cells) locations. For intratumoral signals, attempts were made to classify the expression as tumor cell associated (indicated by the letter “T”), non-tumor cell associated (indicated by the letters “NT”), or both (indicated by “T/NT”). Scores were assigned using a 0–5 semiquantitative scale assessing prevalence of positive cells where 0, negative; 1, minimal or rare; 2, low; 3, moderate; 4, high; and 5, very high. Samples where melanin content was too high to confidently assign a PD-L1 score were specifically noted. (2) Percentage estimates: scores at the low end were given a score of 0 % of tumor cells, <1, 1, and 3 % (roughly indicates >1 % but <5 %). Scores at the high end were given in 10 % increments.

### Statistical analysis

Descriptive statistics were used to tabulate and present the study findings.

## Results

Twenty-four patients where metastatic melanoma presence in the SLN was confirmed by H&E review of the cut sections were included in the final analysis of PD-L1 expression. Table 1 summarizes patient demographics and disease characteristics. Table 2 summarizes the results of tumor associated PD-L1 expression. Three patient samples were noted to have high melanin content where it was difficult to assign a PD-L1 expression score.

**Table 2:** Tumor associated PD-L1 expression

Patient with SLN+ (N = 24)	Tumor associated expression		
	Semi-quantitative scoring		Percentage estimates—tumor
	PD-L1 score—tumor	PD-L1—peritumoral	
1	1 T, very weak	1	3
2	4, predominantly T, peripheral	1	40
3	Melanin confounds	2	NA
4	0	2 (adjacent subcapsular sinus)	0
5	4 T/NT	2	30
6	2, predominantly NT	3	3
7	0	3 (adjacent subcapsular sinus)	0
8	2.5, predominantly NT	3	10
9	4.5, predominantly T, weak	2 (adjacent sinus)	50
10	Melanin confounds	0	NA
11	1 T	2.5	<1
12	0^a^	2	0
13	1 T	1	<1
14	2.5 NT	2	10
15	2 predominantly NT	3	3
16	1 T	2	1
17	Melanin confounds	0	NA
18	1	1	<1
19	1 NT	2.5	<1
20	2T?, melanin confounds	2	<1
21	4, predominantly T, peripheral	3.5	40
22	5, predominantly T	2	90
23	4	0	40
24	4.5 T/NT	0	70

^a^Very few tumor cells. In one region where a few tumor cells were present, a dense area of PD-L1 positivity was present directly adjacent but was impossible to say if all were non-tumor; this is interpreted as most likely

**Fig. 1:**
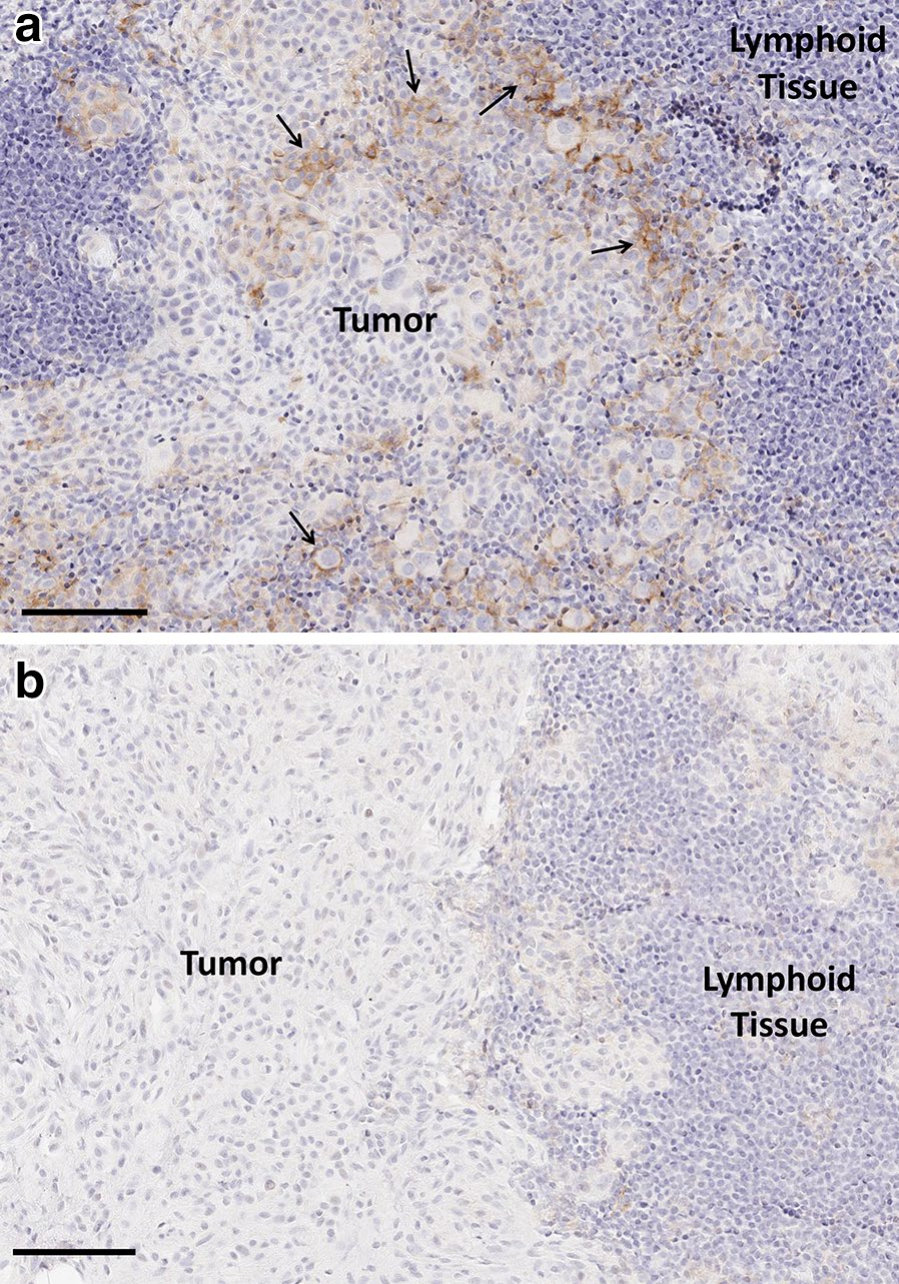
Examples of sentinel lymph node melanoma sample intratumoral PD-L1 immunohistochemical staining, including positive for PD-L1 expression (*brown* chromogen) (**a**) and negative (**b**). The *arrows point* to representative areas of membranous staining interpreted as tumor cell expression. The *scale bar* represents 100 μm

Of the remaining 21 patient samples, 13 (62 %) showed PD-L1 positivity defined as partial or complete membrane staining in ≥1 % (range 1–90 %) of tumor cells using the 22C3 antibody. The majority of patient samples (20 samples; 95 %) showed some degree of intratumoral or peritumoral PD-L1 expression. Table 3 presents the frequency of tumor-associated PD-L1 expression by the percentage of expression. Among patient samples that were considered PD-L1 positive, more than half were scored at >10 %. Figure 1 shows examples of SLN melanoma sample intratumoral PD-L1 immunohistochemical staining, both positive (A) and negative (B). Finally, we attempted to score the non-tumor (lymphoid tissue) associated PD-L1 expression in the 24 patient SLN samples included in this study. PD-L1 expression in these areas was classified as (1) expression by histiocytes in the sinuses of the node and (2) expression observed in non-sinus lymphoid tissue with morphologic characteristics consistent with antigen presenting cell populations (dendritic cells and macrophages). Table 4 summarizes these observations.

**Table 3:** The frequency of tumor-associated PD-L1 expression by percentage of expression (excluding samples where melanin content was too high to confidently assign a PD-L1 score; N = 21)

Percentage estimates	No. of patients (N = 21)	Percentage of patient (N = 21)
0	3	14
<1	5	24
1–10	6	29
>10	7	33

**Table 4:** Non-tumor associated lymphoid tissue PD-L1 expression

Patient with SLN+	Non-tumor associated lymphoid tissue (semi-quantitative scoring method)	
	PD-L1—sinuses	PD-L1—APC pattern
1	4	3
2	2	2
3	4	2
4	4	2.5
5	2	2
6	4	3
7	5	4
8	1	2
9	3	3
10	4	4
11	1	4
12	3	3
13	2	2
14	2	1
15	3	3
16	5	3
17	4 (intense)	3
18	4	2.5
19	3	2.5
20	3	4
21	3	4
22	3	3
23	4	4 (intense)
24	4	4

## Results and discussion

In this study, we have reported that melanoma patients with SLN micrometastases detected by SLN biopsy demonstrate evidence of tumor associated PD-L1 expression. The majority of patients were found to have tumor associated PD-L1 expression estimated by two scoring methods, including the percentage estimates method utilized in prior studies in patients with metastatic melanoma [34]. Inhibition of the PD-1/PD-L1 axis has emerged as an important immune checkpoint therapeutic strategy with unprecedented clinical responses seen in patients with metastatic melanoma. Efforts are underway to test anti-PD1 monoclonal antibodies as adjuvant therapy in patients with operable melanoma who continue to carry a high risk of recurrence and death after surgery. In these patients, residual micrometastatic disease is expected to be the source of future melanoma relapse. For patients with AJCC stages IIB-C/III/IV, melanoma carries a high risk for recurrence and death from melanoma with surgical management alone [35–38]. Systemic adjuvant therapy that targets melanoma micrometastases is indicated postoperatively where it may provide the greatest opportunity for cure before relapse into advanced inoperable stages. Multiple systemic therapeutic agents have been tested as adjuvant therapy for melanoma with durable benefits seen only with high dose interferon-alfa (HDI) to date [39]. CTLA4-blockade with ipilimumab is currently being tested in the adjuvant EORTC 18071 trial (stage III; compared to placebo) and U.S. Intergroup E1609 (stage III and IV; compared to HDI). Ongoing adjuvant trials are also targeting patients with BRAF mutant melanoma including vemurafenib (BRIM-8) and dabrafenib/trametinib (COMBI-AD). Adjuvant trials involving PD1-blockade are expected to be activated in the second quarter of 2015. In this study, we have characterized PD-L1 expression within clinically/radiologically negative but microscopically tumor positive SLN, which may be important to our understanding of the relevance of this immune checkpoint pathway for adjuvant therapy.

The findings of this study further support the investigation of PD-1/PD-L1 blockade in the adjuvant setting in melanoma patients with high risk for relapse.

Previous studies have shown that increased tumor associated expression of PD-L1 is associated with a higher likelihood of clinical response to PD-1/PD-L1 blockade [40–42]. Kefford et al., reported that melanoma patients receiving pembrolizumab in the phase I KEYNOTE-001 trial had differential clinical responses to treatment based on the baseline tumor PD-L1 expression pattern [34]. PD-L1 positivity in pre-treatment biopsies was defined as partial or complete membrane staining in ≥1 % of tumor cells using the 22C3 antibody [34]. Patients with PD-L1 positive tumors had a 49 % overall response rate (ORR) as compared to 13 % in those with PD-L1 negative tumors. In the phase I study that tested multiple doses of nivolumab in advanced melanoma, an exploratory analysis to investigate tumor PD-L1 expression and its association with treatment response was also undertaken [43]. PD-L1 positivity was defined as tumor membrane staining with any intensity and with cut-off values of 1 and 5 %. ORR was superior in patients with a PD-L1 positive status. In the 1 % cutoff analysis, 35 % of patients considered PD-L1 positive had an objective response versus 13 % in PD-L1 negative. The ORR increased (44 %) in PD-L1 positive patients by increasing the cutoff to 5 % with no change in the ORR in the PD-L1 negative group. Overall survival (OS) in PD-L1 positive patients was 25 months as compared to 12 months in PD-L1 negative at 1 % cutoff [43]. By increasing the cutoff to 5 %, median OS was not reached in the PD-L1 positive cohort as compared to 13 months in PD-L1 negative. Similarly, PFS was 9 months in PD-L1 positive patients as compared to 2 months in PD-L1 negative, at both 1 and 5 % cutoff [43]. Similar findings have been reported in other tumor types in patients treated with anit-PD1 antibodies. For example, patients with non-small cell lung cancer (NSCLC) receiving nivolumab in a phase I clinical trial also showed correlation between clinical efficacy and tumor PD-L1 expression [44]. PD-L1 negative tumors showed no objective responses to nivolumab as compared to a 50 % ORR in patients with PD-L1 positive status. PFS at 24 weeks was 70 % and 1-year OS of 80 % in PD-L1 positive patients as compared to 57 and 71 % in PD-L1 negative patients respectively [44]. The extent of PD-L1 expression was also found to correlate with ORR in a recent study of pembrolizumab in NSCLC with shorter progression-free and overall survival among patients with low tumor expression of PD-L1 [42]. Recently, PD-L1 expression in the immune infiltrate in the tumor has also been found to be associated with response to PD-1 pathway blockade therapy [45]. Interestingly, tumor PD-L1 negative status although it carries a lower likelihood of response to anti-PD1 antibodies, it does not preclude response and it is widely understood that the clinical utility of PD-L1 tumor expression requires refinement. However, these observations in patients with metastatic melanoma and other malignancies, support our hypothesis that micrometastatic tumors that are the source of future melanoma relapse in high risk patients express PD-L1 making them susceptible to PD1/PD-L1 therapeutic blockade.

Scoring of non-tumor associated PD-L1 expression in in the SLN was complicated by the fact that historical PD-L1 scoring has consistently centered on expression localized to and immediately surrounding tumor tissue. Normal or reactive lymphoid tissue is not typically included in scoring, so no experiential base exists for this type of analysis. In order to try to provide maximal information however, attempts were made to provide scores of PD-L1 expression in non-tumor infiltrated regions of lymph nodes where metastatic lesions were identified. PD-L1 expression in these areas could be classified as falling into two basic categories: (1) expression by histiocytes in the sinuses of the node and (2) expression observed in non-sinus lymphoid tissue with morphologic characteristics consistent with antigen presenting cell populations (dendritic cells and macrophages). Each of these categories was scored separately using a 0–5 semi-quantitative system, focused on signal prevalence. Given the very different representation of tissue from sample to sample and the lack of historical experience with this type of non-tumor associated scoring by the evaluating pathologists, this data should be interpreted as extremely exploratory, and not suitable for drawing conclusions.

## Conclusions

In conclusion, tumor-associated PD-L1 expression is readily detectable within melanoma micrometastases in the SLN of the majority of patients included in this study. These results support the testing of a therapeutic role for PD1/PD-L1 inhibition in the adjuvant therapy setting, targeting melanoma micrometastases. The testing of PD-L1 expression as a predictive biomarker for therapeutic benefit from anti-PD1 therapy is also warranted and to the best of our knowledge is planned in the context of ongoing adjuvant trials.

## Abbreviations

AJCC: American Joint Committee on Cancer
CTLA-4: cytotoxic T-lymphocyte-associated protein 4
EORTC: European Organisation for Research and Treatment of Cancer
FDA: Food and Drug Administration
HDI: high dose interferon-alfa
H&E: hematoxylin and eosin
IHC: immunohistochemistry
ORR: overall response rate
OS: overall survival
PFS: progression free survival
PD1: programmed cell death protein 1
PD-L1: programmed death-ligand 1
SLN: sentinel lymph node
FFPE: formalin-fixed, paraffin-embedded
